# The Rare Case of an Adult-Onset Xanthogranuloma of the Paranasal Sinuses: A Histological Dilemma

**DOI:** 10.1155/2020/2847821

**Published:** 2020-06-08

**Authors:** J. Bastianpillai, N. Haloob, S. A. Panchappa, J. Marais

**Affiliations:** Department of ENT, Northwick Park Hospital, UK

## Abstract

Xanthogranuloma is a rare benign tumour, part of the non-Langerhans cell histiocytosis group, uncommon in adults and even less common in the paranasal sinuses. Despite its benign nature, it mimics neoplasm due to its local effects which can have serious functional consequences depending on the anatomical location. We present the rare case of a young lady who presented insidiously with a maxillary sinus xanthogranuloma and was treated with endoscopic resection. Tissue diagnosis is of paramount importance to guide correct further investigations and management, and we discuss the potential challenge in identifying such a rarely seen pathology.

## 1. Introduction

**Figure 1:**
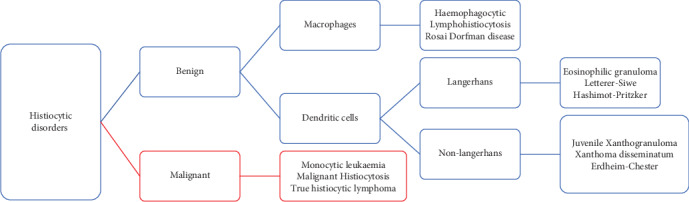
Classification of histiocytic disorders based on cell origin (adapted from Li et al.).

Histiocytes are a type of phagocyte derived from dendritic cells or macrophages and are produced in the bone marrow. These cells can travel haematologically to distant tissues with the potential to cause pathological proliferation and deposition, resulting in local tumour formation [1]. Several classification systems for histiocytic disorders exist; however, the most widely accepted is by the World Health Organisation (WHO) which categorises them into three groups as Langerhans cell histiocytosis (LCH), non-Langerhans cell histiocytosis (non-LCH), and malignant histiocytosis [2]. Within these groups are multiple disease subtypes, and xanthogranuloma falls within the non-LCH group (Figure 1) and is uncommonly found at extracutaneous sites. It is a benign condition with a good prognosis and is not typically invasive [1].

Due to the variable anatomical involvement, clinical presentation and diagnosis can be challenging and is largely based on detailed specialist histological analysis.

We present the rare case of a young lady who presented with nasal symptoms and a unilateral sinonasal mass, which was found to be an adult-onset xanthogranuloma.

## 2. Case Presentation

A thirty-five-year-old Caucasian lady was referred by her GP to General ENT clinic after presenting with a mechanical fall and head injury, who then developed a sudden onset anosmia, ageusia, and worsening right-sided nasal obstruction and discharge, which was occasionally blood-stained. This was associated with right-sided facial pain which radiated to her right ear and perioral region. She was otherwise systemically well. She had no significant past medical history, took no regular medication, was a nonsmoker, and worked as a journalist with travel to Rio de Janeiro and rural Uganda in the preceding two years.

Flexible nasendoscopy revealed what appeared to be an enlarged and oedematous right middle turbinate obstructing the middle meatus, with surrounding mucopus tracking back to the postnasal space. Remaining head and neck examination including cranial nerves was unremarkable.

**Figure 2:**
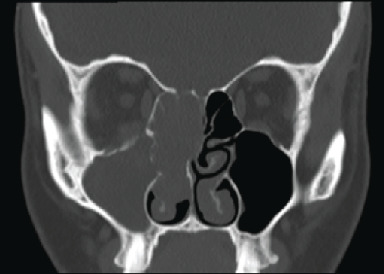
Contrast-enhanced CT sinuses (coronal section). Shows right-sided lesion within the nasal cavity associated with bony erosion and slight deviation of the upper aspect of the nasal septum to the left. There is associated complete soft tissue opacification of the frontal sinuses, ethmoidal sinuses, and maxillary sinus. Note the close relationship with the anterior ethmoid artery.

An urgent contrast-enhanced CT scan of the sinuses was performed (Figure 2) which showed a mass lesion within the right nasal cavity causing local expansion and deviation of the nasal septum to the left and bowing of right medial orbital wall with opacification of the frontal, ethmoid, and maxillary sinuses and bony demineralisation of the adjacent skull base. The medial orbital wall appeared intact and the left sinonasal spaces and postnasal space were unremarkable.

**Figure 3:**
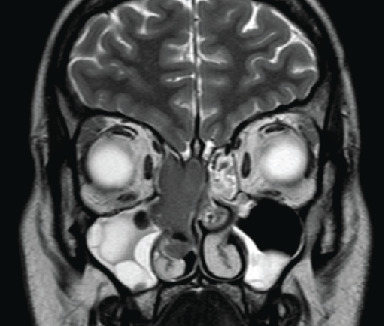
Contrast-enhanced MRI sinuses (coronal section, T2-weighted image). Demonstrates the demarcation between the mass and polypoid maxillary sinus, not appreciated on CT.

**Figure 4:**
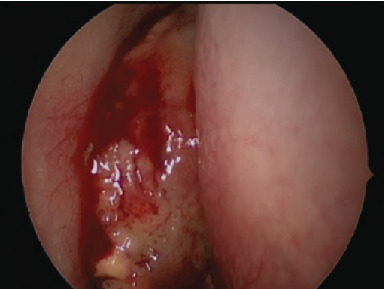
Intraoperative endoscopic photograph displaying the soft tissue mass in the right nasal cavity obstructing the middle meatus.

Further evaluation with a contrast-enhanced MRI scan of the sinuses (Figure 3) demonstrated a lobulated right nasal cavity soft tissue mass involving the middle turbinate and uncinate process and extending superiorly making contact with the cribiform plate and olfactory recess but not extending intracranially. There was secondary frontal and maxillary sinus obstruction. The mass showed homogenous mild uniform enhancement (visualised best on T2-weighted images). Appearances were concerning for an aggressive malignant lesion, subsequently leading to urgent diagnostic endoscopic resection (Figure 4). Intraoperatively, the mass occupied the area of the right middle turbinate and anterior ethmoid air cells. The tumour was very friable hence removed piecemeal, with biopsies sent for histology. This was achieved with the aid of zero-degree and thirty-degree endoscopes. Both the medial orbital wall and skull base were exposed and remained intact. Ethmoidectomy, sphenoidotomy, and frontal recess clearance were performed, and a large middle meatal antrostomy was created which revealed copious mucous. Floseal® and NasoPore® were inserted into the nasal cavity, and she was started on a saline douche postoperatively.

**Figure 5:**
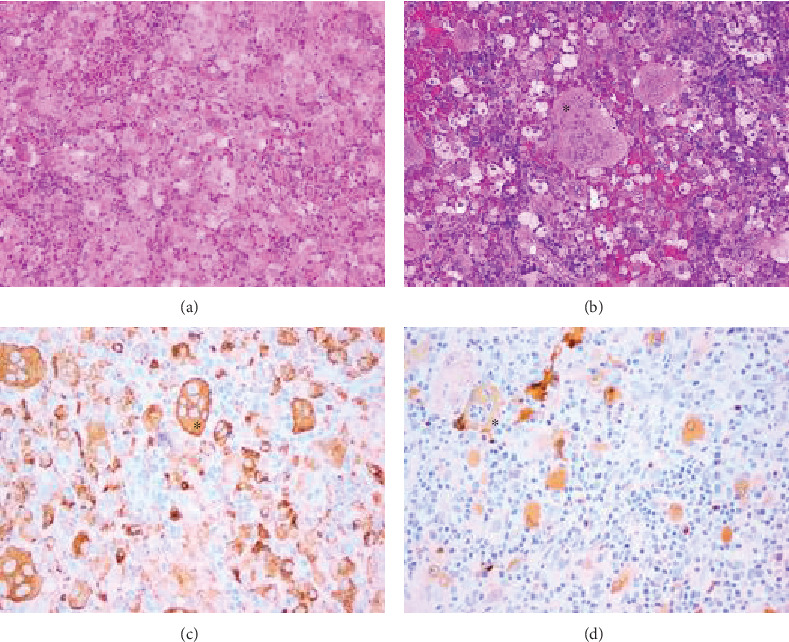
(a) Haematoxylin and eosin stain ×100 magnification: showing sheets of histiocytes with abundant eosinophilic cytoplasm intermingled with polymorphs. (b) Haematoxylin and eosin stain ×100 magnification: showing multinucleate giant cells in the infiltrate (∗). (c) CD68 stain ×200 magnification: highlighting histiocytes (∗). (d) S100 stain ×200 magnification: a giant cell shows engulfed polymorphs in its cytoplasm (∗).

The histology (Figure 5) showed a polypoid lesion composed of a proliferation of large foamy histiocytic cells with associated lymphocytes, neutrophils, and eosinophils. The histiocytes had variable morphology and immunohistochemistry revealed expression of CD45, CD163, and CD68 and patchy positive staining of S-100 and CD1a. Although there was no evidence of malignancy, an appropriate histopathological diagnosis could not be reached following specimen review by several local centres, and expert opinion was eventually sought from a histopathologist in Italy. Possible diagnoses included rhinoscleroma, Langerhans cell histiocytosis, and Rosai-Dorfman disease; however, the results of microbiological studies and mutational analysis had, respectively, excluded these. It was concluded that the features were thought to be most consistent with xanthogranuloma.

**Figure 6:**
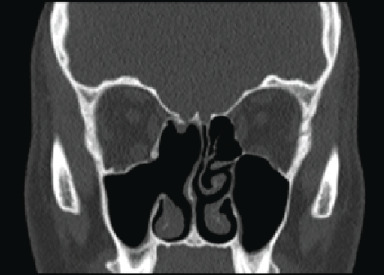
Noncontrast enhanced CT sinuses (coronal section). Postoperative scan demonstrates right ethmoidectomy, middle turbinectomy, and creation of middle meatal antrostomy. Well-aerated sinonasal spaces and no tumour recurrence.

She remained well and asymptomatic one year on from her excision, and follow-up imaging was reassuring of no disease recurrence (Figure 6).

## 3. Discussion

Non-Langerhans histiocytic diseases are poorly understood and diagnosis is challenging due to the multiple inconsistent definitions of the disease in the literature. The Histiocyte Society provided the clearest classification system in 1987, which was subsequently updated in 1997 by the WHO (Table 1) [2]. There has since been a more complex classification proposed by Emile et al. based on a number of histological and molecular factors [7]; however, for simplicity, we shall refer to the established WHO classification. Class 1 and 2 histiocytic disorders comprise benign pathology, and Class 3 is associated with malignancy. In general, the initial clinical presentation can be similar between the subtypes but may differ in anatomical site, progression, and hence management.

Differentiation between the various classes and subtypes is made histologically with immunohistochemistry as first identified by Weitzman and Jaffe [8]. Class 1 disorders typically feature overproliferation and deposition of dendritic cells called Langerhans cells that demonstrate positivity to CD1a and S-100. Conversely, if these markers are absent from the dendritic cells on staining, they are then recognised as non-LCH.

Xanthogranuloma, a non-LCH, is typically a disease of childhood and adolescence, known as juvenile xanthogranuloma (JXG), manifesting as cutaneous lesions predominantly in the head and neck, but less commonly the trunk and extremities. Compared to other non-LCH variants, JXG lesions are often solitary [9]. Although rare, extracutaneous organ involvement is possible, with the orbit and testes having been reported [10, 11]. Xanthogranuloma was first described in 1905 by Adamson who observed cutaneous nodules in infancy, though the term JXG was proposed in 1954 by Helwig and Hackney [12, 13].

Adult-onset JXG was first reported in 1963 by Gartmann and Tritsch, and since then, it is estimated that 10% of JXG is found in adults with an equal sex distribution and median age of 35 years [8, 14]. Despite being a proven clinical phenomenon in adults, it is still confusingly referred to as JXG in many parts of the literature, with a few sources coining terms such as adult-onset xanthogranuloma, solitary or multiple adult xanthogranuloma or late-onset JXG [9].

The lesions of adult-onset xanthogranuloma are similar to JXG; however, they may be larger and do not present with hyperlipidaemia, in contrast to JXG, as was the case in this patient. Whilst histiocytosis can be multicentric, this is not typically seen in adult xanthogranuloma, and therefore systemic medical management with immunosuppressive or corticosteroid therapy is usually not indicated. Furthermore, the rate of disease regression is reportedly 54% in adult disease compared to 83% in JXG, though the prognosis is remarkably favourable [15].

Xanthogranuloma in the paranasal sinuses is not a widely reported phenomenon with only a handful of cases in the English literature. Symptomology depends on the sinonasal site involved, but typical clinical features include nasal obstruction, epistaxis, and focal pain. It is sometimes regarded as part of a disease group known as “inflammatory pseudotumours” [16]. Though this vague term is commonly used in the literature, it offers little practical benefit, as it represents any nonspecific, chronic expanding inflammatory lesion clinically or radiologically mimicking a neoplasm. Histological analysis is necessary for correct tissue subtyping in order to guide correct patient management. Of particular note, in this case, was the difficulty in establishing a tissue diagnosis given the unusual histological characteristics. That it required input from several centres before a diagnosis was established, meant that further treatment planning was delayed and was a great source of anxiety for the patient.

Given the benign nature of the disease, management is usually symptom-driven, and the gold standard is surgical resection; however, complete resection may not be achieved if key anatomical structures are at risk such as the orbit or skull base and such cases require the review of a head and neck multidisciplinary team. Whilst chemo- and radiotherapy have reportedly been used in LCH, the role of this is not fully established in non-LCH [17].

On reflection of the presentation of this case, it is likely that her previous head injury coincided with progression of the mass lesion. Nevertheless, the diagnostic process proved to be a challenge to both surgeons and histopathologists requiring exclusion of other more common conditions affecting this anatomical region such as Rosai-Dorfmann disease or rhinoscleroma through specialist immunohistochemistry, microbiology, and mutational analysis. Rosai-Dorfmann disease is a benign histiocytic disorder, derived from a macrophage lineage, and may clinically present in a similar fashion to xanthogranuloma but is typically associated with massive lymphadenopathy and “B” symptoms. It also expresses positivity to S-100, CD68, and CD163 but is negative for CD1a [3, 4]. Rhinoscleroma is a chronic granulomatous condition that may also present as a maxillary sinus mass but is associated with *Klebsiella rhinoscleromatis* infection [5, 6]. It is therefore important to recognise this disease entity within the scope of sinonasal disease although it remains very rare.

### 3.1. Learning points

- Xanthogranuloma is a benign type of histiocytosis rarely seen in adults and rarely affects the sinonasal cavities
- Its presentation within the sinonasal cavity often clinically and radiologically mimics that of a neoplastic process which should be ruled out in the first instance
- Histiocytic disease encompasses a spectrum of disorders which have variable clinical presentation and course, and hence subtype diagnosis requires detailed histopathological analysis with immunohistochemistry staining.
- Surgical resection is the mainstay of treatment and symptom relief but with careful consideration to surrounding anatomical structures.

## Figures and Tables

**Table 1:** WHO classification of histiocytic disorders (adapted from Favara et al.).

**Class 1:** dendritic cell histiocytoses
Langerhans cell histiocytosis Erdheim-Chester disease Juvenile xanthogranuloma
**Class 2:** nondendritic cell histiocytoses
Rosai-Dorfmann disease Reticulohistiocytoma
**Class 3:** malignant histiocytoses
Acute monocytic leukaemia Malignant histiocytosis True histiocytic lymphoma
